# Rare Pathology Case Report: Low-Grade Endometrial Stromal Sarcoma Forming Sex Cord– and Endometrioid Gland–Like Differentiation in Metastatic Foci

**DOI:** 10.1155/2024/4073869

**Published:** 2024-09-04

**Authors:** Haneen Al-Maghrabi, Ghadeer Mokhtar, Jaudah Al-Maghrabi

**Affiliations:** ^1^ Department of Pathology and Laboratory Medicine King Faisal Specialist Hospital and Research Center, Jeddah, Saudi Arabia; ^2^ Department of Pathology Faculty of Medicine KingAbdulaziz University, Jeddah, Saudi Arabia

**Keywords:** endometrial gland–like differentiation, endometrial stromal sarcoma, metastasis, sex cord–like differentiation

## Abstract

Low-grade endometrial stromal sarcomas (LGESSs) are indolent tumors with a slow progression rate that tend to recur locally. They represent up to 10% of all primary sarcomas of the uterus and endometrium and only 0.2% of all genital tract tumors. They are commonly present in a younger demographic compared to other uterine tumors, with patients' ages typically between 42 and 58 years old. Although the overall 5-year survival rate is excellent, it has a natural history of delayed metastases which may manifest even decades after the disease was first diagnosed. They typically present as poorly defined lesions infiltrating the myometrium, along with extensive engagement of surrounding vascular structures. LGESS may display variants of different morphologies such as smooth muscle, fibromyxoid, sex cord-like, and endometrioid-type gland differentiation. These variations can pose a diagnostic challenge. The occurrence of this differentiation in a metastatic focus rather than in the primary tumor is seldom recorded in the literature. We present a case of a 51-year-old lady with a history of LGESS who was treated with surgery and radiotherapy and then presented after 12 years with an inferior vena cava (IVC) mass, which was confirmed histologically to be metastatic LGESS. Immunohistochemistry studies reveal strong positivity for CD10, WT1, and PR. These markers were negative in the sex cord and endometrioid gland–like differentiation counterparts. The patient had her initial follow-up appointment after the IVC mass resection, and she was in good health with no complications. To the best of our knowledge, this case represents a unique instance of metastatic LGESS exhibiting both sex cord and endometrioid gland–like differentiation that has not been observed in the primary tumor.

## 1. Introduction

Uterine mesenchymal neoplasms encompass various types of tumors such as smooth muscle tumors, endometrial stromal tumors (ESTs), homologous and heterologous sarcomas, sarcomas of uncertain pathogenesis, and mixed tumors that involve both epithelial and mesenchymal cells including the group of uterine tumors resembling ovarian sex cord tumor (UTROSCT). Although ESTs are the second most prevalent among these tumors, they are still comparatively infrequent. Low-grade endometrial stromal sarcoma (LGESS) comprises only 1% of all malignant uterine tumors and 10% of all uterine sarcomas. The age range is considered widely distributed, but there is a preference for perimenopausal ladies with an overall average age of 52 years [1]. Extended utilization of estrogen or tamoxifen and exposure to pelvic radiation are considered risk factors. LGESS is a well-known tumor for its ability to persist and metastasize even several years after it has been initially removed. Commonly, LGESS is characterized by a high prevalence of proliferative differentiation, such as smooth muscle, fibroma-like, fibromyxoid, glandular endometrioid, pseudopapillary, sex cord, clear cell, epithelioid, rhabdoid, adipocytes, or myxoid differentiation within the tumor [2]. Several potential pitfalls encompassing both benign and malignant differentials exist. It is worth mentioning that LGESS with sex cord element and endometrioid gland–like differentiation is rarely reported solo in a metastatic deposit. In our case report, the initial instance of metastatic LGESS to the inferior vena cava (IVC) manifests sex cord–like and endometrioid gland–like differentiation, which was not evident in the primary resected tumor. Additionally, we have conducted a relevant literature review.

## 2. Case Presentation

**Figure 1:**
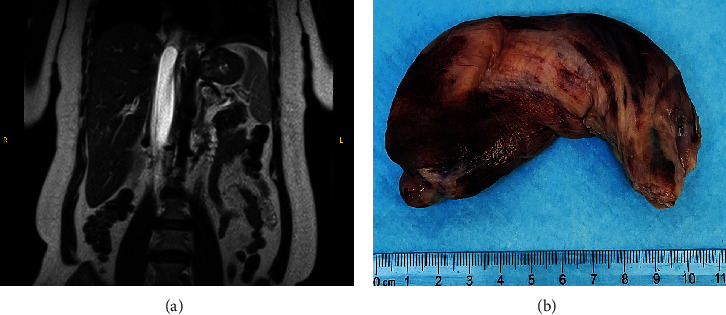
Radiology and gross tumor examination. (a) T2 magnetic resonance imaging (MRI) shows extensive venous thrombus extending from the right internal iliac vein to the right atrium, representing tumoral thrombosis or an intravenous mass inferiorly with transition into bland thrombus at the level of the intrahepatic inferior vena cava (IVC). (b) A gross examination of the mass measures around 10 cm.

**Figure 2:**
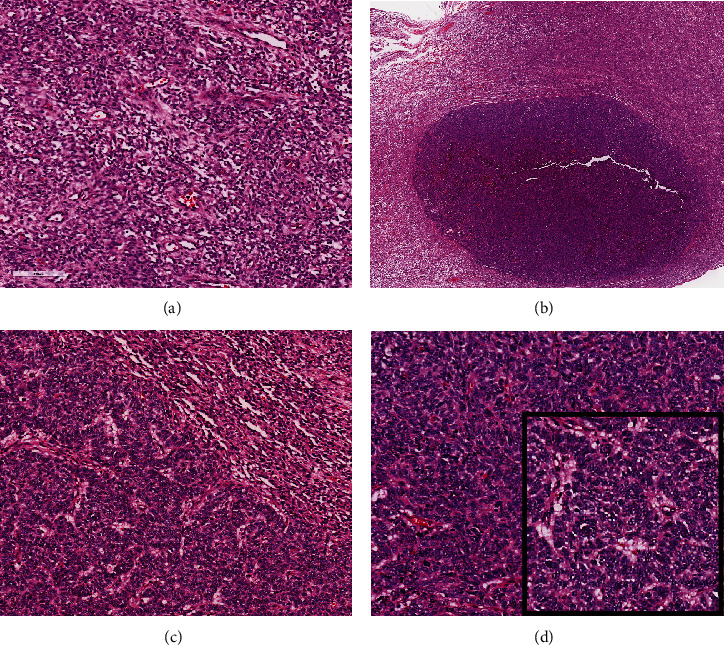
Histopathology examination of the tumor by hematoxylin and eosin (H&E) stains. (a) Tumor section taken from the primary surgical resection (original hysterectomy specimen) that shows bland spindle cell proliferation with whorling around small blood vessels (H&E; 20×). (b) Microscopic evaluation of metastatic LGESS with a focal area of differentiation formed as a distinctive nodule (note the sharp demarcation) (H&E; 2×). (c) Focal areas of the tumor composed of cellular and compact branching cords and trabeculae of tumor cells representing sex cord–like differentiation (H&E; 20×). (d) Clusters of epithelioid cells arranged in glandular-like differentiation with variable cytoplasmic amount, round nuclei, and minute nucleoli arranged in tubules (H&E; 20×). Note the occasional nuclear groove (inset) (H&E; 40×).

**Figure 3:**
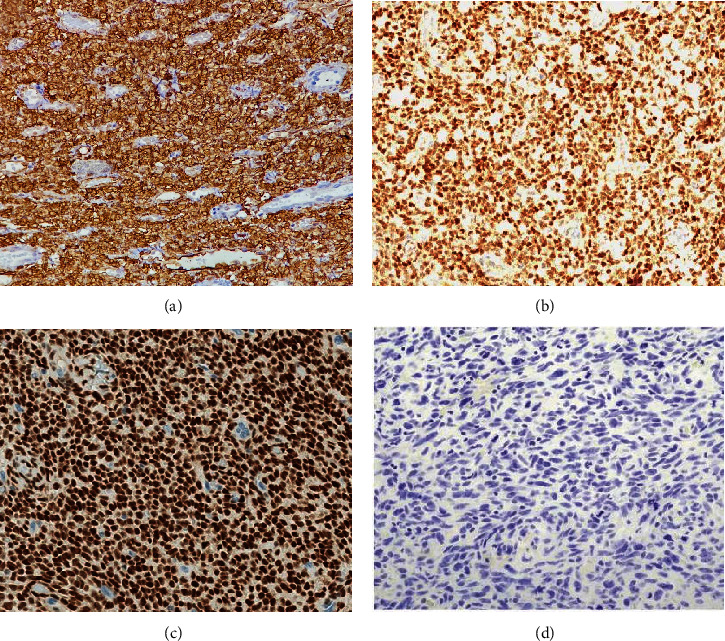
Tumor examination by confirmatory immunohistochemistry studies. (a) Tumor cells are strongly immunoreactive for CD10 (expressed in both conventional areas and areas of glandular differentiation) (40×). (b) The tumor cells show strong positivity for WT1 (40×). (c) Clear nuclear positivity for estrogen receptor (expressed in both conventional areas and areas of glandular differentiation) (40×). (d) The tumor cells are completely negative for Cyclin-D1 immune staining (40×).

A 51-year-old lady with a history of total hysterectomy and right salpingo-oophorectomy for the past 12 years was diagnosed with LGESS. The tumor was invading more than half of the myometrial thickness of the adjacent right fallopian tube, and ovary invasion along with extensive vascular invasion was noted at that time. During the initial diagnosis, a pathology examination indicated that the tumor had a low-grade histology. No high-grade endometrial stromal sarcoma (HGESS) was found, and there were no areas exhibiting any other type of differentiation. She was treated with surgical resection followed by radiotherapy at the time of diagnosis. The patient's latest follow-up at the gynecology clinic was 2 years ago, and her chest, abdomen, and pelvis computerized tomography (CT) scan revealed no signs of disease recurrence, indicating a positive outcome. A few months prior to her current presentation, the patient complained of generalized fatigue, body ache, and shortness of breath. Her symptoms were on and off, moderate to severe, and lasted for a couple of months. Magnetic resonance imaging (MRI) of the abdomen and pelvis shows dilated right internal iliac and right common iliac veins with high T2 and low T1 structures extending into the IVC and right atrium, involving the distal part of the left renal vein for 1.2 cm. It spares the right external iliac, left common iliac, right renal, and hepatic veins. It shows enhancement and diffusion restriction in its proximal and mid-region, which is more prominent inferiorly. The restriction and enhancement become subtle at the level of suprarenal IVC with a lack of diffusion restriction at suprahepatic IVC and right atrium (Figure 1(a)). The patient underwent an IVC tissue biopsy by interventional radiology. The histopathology result of the tissue biopsy was consistent with metastatic LGESS. The case was discussed in a multidisciplinary tumor board meeting and decided to do surgical resection of the mass. Intraoperatively, a large mass was found extending from the IVC to the right atrium and ventricle. The mass was muscular in nature. There were no clots around it. There was no direct attachment of the mass to the right atrial wall, right ventricle, or tricuspid valve. After tumor resection, the right atrium was closed, and a chest tube was inserted and closed in the usual fashion. The patient was removed to the recovery room, and she was vitally stable. The resected mass was sent for histopathology examination which grossly measured around 10 cm in maximum dimension (Figure 1(b)). Histopathology examination shows monotonous oval to spindle tumor cells containing minimal to no cytologic atypia, vesicular chromatin, and a scant amount of cytoplasm. No areas of necrosis were seen. Interestingly, one microscopic focus shows a well-demarcated, more cellular area composed of compact tumor cells with nested growth patterns, exhibiting both features of glandular- and sex cord–like differentiation (Figure 2). This distinct area was even characteristic of immunohistochemistry staining expression, including inhibin, calretinin, cluster of differentiation (CD) 99, melanoma antigen recognized by T-cells (MART-1) or MelanA, and Wilms's Tumor-1 (WT1) in the sex cord–like area. The endometrial glands exhibited positive reactivity for cytokeratin and paired-box gene-8 (PAX-8), along with a very faint and weak expression of epithelial membrane antigen (EMA) in the context of glandular-like differentiation. The rest of the ESS cells were strongly reactive for CD10, WT1, estrogen receptor (ER), and progesterone receptor (PR), while totally negative for Cyclin-D1 (Figure 3). Extensive tissue sampling and regrossing of the specimen reveal no other foci of differentiation. No molecular/cytogenetic tests were conducted on this case because of the known previous history, typical cytomorphology, and matching immunohistochemistry result studies. Finally, the case was discussed in the tumor board, and the recommendation was to start her on a hormonal therapy–aromatase inhibitor for 3–4 months duration, along with close clinical follow-up every 2–3 months, and to repeat a CT scan every 4 months. The patient was seen for her first follow-up, 1 year after the surgery, and she was healthy and had no complaints.

## 3. Discussion

ESTs are uncommon and fascinating mesenchymal neoplasms of the uterus that exhibit diverse histopathological, immunohistochemical, and molecular features. These tumors bear a resemblance to endometrial stromal cells during the proliferative stage of the menstrual cycle. In 1966, Norris and Taylor categorized ESTs into benign and malignant classifications based on the mitotic count [3]. The fifth edition of WHO-2020 has classified ESTs into four categories, such as endometrial stromal nodules (ESNs), LGESSs, HGESSs, and undifferentiated uterine sarcomas (UUSs) [4]. Patients with LGESS may present with abnormal uterine bleeding or clinical signs and symptoms associated with extra uterine/pelvic involvement. It is more frequent for these tumors to affect the uterine corpus rather than the cervix. Grossly, it manifests as several indistinct, frequently merging yellow nodules within the endometrial cavity and myometrium. Microscopic examination reveals extensive myometrial invasion in irregular islands along with numerous vascular invasions. Nuclear morphology reveals elongated oval to spindle bland cells, like endometrial stroma, during the proliferative phase. A low occurrence of mitotic activity (less than five per 10 high power fields) is typical, although it may be higher. Additionally, there is no tumor necrosis. The tumor cells are often observed to grow around a swirling pattern of small arterioles and capillaries in a rich vascular network stromal background, which may have hyalinized walls. Additionally, tumor cells can be found mixed with collagen bands, plaques, and histiocytes, either individually or in small groups. Various types of microscopic tumor differentiation features can be observed in the tumor, and these include smooth muscle, fibroblastic, epithelioid, rhabdomyoblastic, myxoid, sex cord-like, endometrioid glands, clear cells, granular cells, bizarre cells, rhabdoid cells, adipose tissue, and papillary/pseudopapillary growth [2]. The tumor cells usually exhibit positive immunohistochemical staining for CD10, WT-1, interferon-induced transmembrane protein-1 (IFITM1), ER, and PR. Cytokeratin expressions are evident in both conventional regions as well as in regions featuring sex cord-like and glandular differentiation. Inhibin, calretinin, CD99, and MelanA are markers that may show positivity in areas with sex cord differentiation. In regions where smooth muscle differentiates, desmin and h-caldesmon are commonly found to be positive. Sometimes, there may be instances of positivity for desmin in conventional regions or regions that exhibit sex cord differentiation. It is uncommon for LGESS to exhibit cells with clear cytoplasm; these cells can exhibit positivity for human melanoma black-45 (HMB45) [2].

While these tumor differentiations have been observed in the primary tumor, they are not commonly seen in metastatic growths that were not initially detected in the main original tumor. We found one case that reported metastatic LGESS to the liver and peritoneal cavity forming sex cord and smooth muscle differentiation, with a history of ESTs forming smooth muscle mixed with sex cord–like differentiation in the main lesion [5]. Another case was reported where LGESS with differentiation resembling sex cord–like elements was found to have metastasized to the thoracic spine. However, it remains unclear whether the original tumor that was resected during total abdominal hysterectomy also had this differentiation or not [6].

In 1976, uterine tumors featuring sex cord–like elements were initially described by Clement and Scully [7]. They described these tumors in which sex cord–like elements made up to 10%–40% of total tumor volume. Sex cord–like differentiation in LGESS shows similarities to ovarian granulosa cell or sertoli cell tumors, forming clusters of epithelioid cells with a variable cytoplasmic amount, round nuclei, minute nucleoli, and low mitosis, arranged in tubules, cords, nests, or trabeculae. Occasionally, nuclear groove or retiform tumor growth can be observed. The required percentage of sex cord–like differentiation in LGESS varies, which is not thoroughly specified in existing literature [8]. Yet due to the deficient reported cases of sex cord and endometrioid gland–like differentiation in metastatic deposits and not in the original resected tumor, the exact pathogenesis behind this pathological finding is not well understood. The differential diagnosis in this case includes HGESS, UTROSCT, and adenosarcoma. HGESS is characterized by high-grade cellular morphology, cellular atypia, and an elevated number of mitotic figures. The cellular component of the tumor contains high-grade round or spindle cells with occasionally accompanied low-grade components [9]. Moreover, areas of tongue-like invasion, tumor cell necrosis, and lymphovascular invasion are commonly detected. Cyclin-D1 and BCL6 transcriptional corepressor (BCOR) immunohistochemistry are commonly observed, while ER is usually absent [10]. Contrary to LGESS, these tumors usually harbor YWHAE-NUTM2A/B fusion [11, 12]. UTROSCT typically lacks any areas of endometrial stroma element component. These tumors show histomorphologic features such as nests, cords, and trabeculae resembling sex cord tumors of the ovary, along with the immunophenotype, which is characterized by the coexpression of epithelial, smooth muscle, sex cord markers, and steroid receptors. These tumors are characterized by ESR1 genetic mutations or GREB1 gene fusions in a subset [13, 14]. Finally, adenosarcoma tumors are composed of bland epithelial cells and condensed periglandular stroma with atypia and increased mitotic activity that form glandular structures resembling a leaf-like structure. They show no sex cord elements [15].

## 4. Conclusion

To conclude, we reported a rare case of LGESS presented with metastasis to IVC reaching the right atrium after 12 years of surgical resection and radiotherapy treatment. As far as we know, this case is an occurrence that has not been reported before of sex cord and endometrioid gland–like differentiation within a metastatic LGESS. This differentiation was not found in the original resected tumor, which was confirmed after reviewing her slides. While the initial radiology examination showed intravascular mass extending from the right iliac vein into the right atrium proposing the possibility of thrombus, tissue sampling was required to confirm the final diagnosis. The presence of sex cord differentiation within metastatic LGESS is not well reported in the literature. We found only two prior cases with sex cord elements present in a metastatic focus, one of which was reported in the main original mass.

## Data Availability

Data is available upon request due to privacy/ethical restrictions.
